# Key Steps in the Evolution of Mammalian Movement: A Prolegomenal Essay

**DOI:** 10.1016/j.neuroscience.2020.05.020

**Published:** 2020-12-01

**Authors:** Robert M. Brownstone

**Affiliations:** Department of Neuromuscular Diseases, UCL Queen Square Institute of Neurology, London WC1N 3BG, UK

**Keywords:** notochord, ventricular zone, sympathetic nervous system, endothermy, spinal cord, movement, microcircuits, cardiovascular evolution

## Abstract

Rich repertoires of movements underlie the complex social interactions of mammals. The building blocks, or syllables, of these movements are produced by spinal cord circuits that are comprised of diverse neuronal types that control musculoskeletal systems comprised of multi-segmented limbs. Together, these systems provide mammals with the evolutionary advantages of power, speed, and endurance. Here, I propose that the key steps in chordate evolution that led to these traits began with the development of the notochord and a proliferative ventricular zone (with associated Notch signalling). This step led to the production of diverse neuronal types that included the development of a sympathetic nervous system that could regulate the evolving cardiovascular system. And the sympathetic nervous system in turn led to the development of homeothermic endothermy, a requirement for motor systems to produce a combination of power, speed, and endurance. Furthermore, the evolution of the continuous structure of the spinal cord led not only to a structure fit for cartesian signalling molecules, but also to one with high processing power in which circuits for effecting movement syllables formed. These syllables are harnessed by higher regions of nervous systems to produce the complex movements required for interactions with others and with the surrounding environment.

## Highlights

• Several evolutionary steps led to the emergence of vertebrate movement needed for social behaviour.
• Descending systems rely on diverse spinal cord neurons to produce a rich repertoire of movement syllables.
• The notochord and ventricular zone led to this diversity of neuronal types.
• The sympathetic nervous system provided the substrate for homeothermic endothermy.
• Homeothermic endothermy is a necessary building block for power, speed, and endurance.

## Preamble

For this issue of Neuroscience dedicated to the scientific contributions of Tom Jessell, I initially fretted about the topic to present. Over two decades of collaboration with Tom, he and I had countless discussions about spinal cord development and motor circuits, including discussions about agnathans, fish, chicks (occasionally), rats, cats (often), and of course mice. So it is interesting that one topic that to my recollection we did not discuss was the evolution of movement. This seems improbable given the breadth of our intense conversations that would often last many hours and during which I could become so engaged that, for example – and only once – I drove right through a stop sign putting his life (not to mention those of our life partners Jane and Liz) at risk. Here, I have attempted to put some thoughts together about the overall success of the evolution of movement with a focus on the spinal cord, knowing that this paper would be far different had Tom been around for healthy debate.

I dedicate this paper to my friend and colleague, Tom Jessell.

## Introduction

On a recent trip to Kenya, I was astonished by the diversity in the animal kingdom, most evident in birds and mammals due only to my focus. In particular, it was astounding to see the vast differences in social structures of the various mammals. These social structures arise from the different paths of the evolution of their brains. But *how* each animal interacts with another and with the environment depends entirely on their motor systems, which are remarkably diverse between species. Despite divergence of mammals over the last ∼100 million years (Springer et al., 1997), the basic building blocks of movement remained more or less the same (Grillner and El Manira, 2020). That is, behaviour is comprised of the concatenation of movement syllables (Wiltschko et al., 2015, Markowitz et al., 2018) that form a common basic vocabulary that expanded through evolution.

One example of movement is locomotion, in which spinal cord circuits produce the rhythm and pattern of movement. This act of progression is necessary for predator and prey, but also ultimately forms the basis of social interactions. Neural circuits for chordate locomotion are old: some invertebrate chordates, such as amphioxus, produce swimming via rather simple circuits (Lacalli and Candiani, 2017). Locomotor circuits became more intricate in the earliest vertebrates that produced undulatory movements over 500 million years ago (mya), further increasing in complexity with development of limbs (initially pectoral fins, ∼420 mya, then tetrapods ∼385 mya; (George and Blieck, 2011, Shubin et al., 1997, Pyron, 2011), and continuing to get more complex with the development of dexterity (perhaps a few mya, depending on definition; evolving convergently in birds and mammals). To understand the “success” of mammals and their social interactions, it is thus necessary to determine the key required steps from early chordate evolution, over 500 mya, to the evolution of multi-segmented limb control in mammals (∼225 mya) (Lucas and Luo, 1993).

The neural circuits that ultimately produce the fundamental syllables of limb movements and ensure that both intralimb and interlimb patterns of muscular contraction are effective for the task at hand are inherent to the spinal cord. The above question related to understanding success can thus be reduced to: what drove the early evolution of the spinal cord? And what were the evolutionary advantages of the spinal cord that ultimately led to the success of vertebrates?

**Fig. 1:**
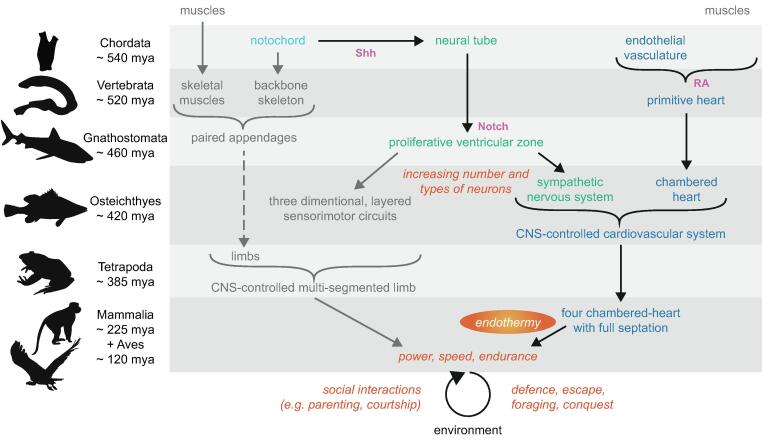
Schema of the key evolutionary steps that led to movements needed for the success of mammalian movement and the timeline (left). Blue: cardiovascular evolution. Green: key neural evolutionary steps. Grey: important steps not discussed to any great detail in this essay. Orange: results of the steps. Pink: some key signalling molecules involved in these steps.

Key developments for the success of vertebrates were power, speed, and endurance. In this brief article, which I hope is simply prolegomenal to further concepts along this vein, I will argue – as a physiologist rather than as an evolutionary biologist (a subject in which I have no expertise) – that these developments were possible because of the evolution of the notochord, neural tube, and ventricular zone (VZ). The VZ provided the substrate for an increase in the number and types of neurons. This neuronal diversity led in turn to, amongst rich sensory and motor circuits, the sympathetic nervous system (SNS). And the SNS laid the foundation for the evolution of homeothermic endothermy (Fig. 1). It was upon this backbone, so to speak, that power, speed, and endurance (and ultimately dexterity) were built. And these traits ultimately supported the development of the brain, such that complex social structures could evolve to make use of the spinal cord circuits that create movement syllables. And that, in an anthropomorphic view, is an evolutionary success story that can be witnessed across the plains of Africa.

## From notochord to ventricular zone

My focus will be on transitions beginning in early chordates and focussing largely on mammals. For discussion about the transition from invertebrates to chordates (see (Holland et al., 2015)). Furthermore, I acknowledge that there is little linearity in evolution, and that each of the key steps that I discuss below involves many additional concurrent processes. I am illustrating this thesis briefly using straight arrows between discrete steps for clarity.

The notochord, of course, appeared with the emergence of the phylum (or superphylum, see (Satoh et al., 2014)) chordata. Each of the 3 subphyla (or phyla), amphioxus, tunicates, and vertebrates, have notochords. In amphioxus, tunicates, and the earliest vertebrates (agnathans including hagfishes and lampreys), the notochord persists through life, whereas in all other vertebrates (beginning with gnathostomes, evolving ∼460 mya) it is transient, existing only in early development (Annona et al., 2015). Nonetheless, it plays a key role in development.

Developmental studies of annelids have revealed that the notochord likely arose from the axochord – a midline muscular structure with molecular characteristics similar to those of the chordamesoderm – developmental midline cells that are the precursor of the notochord (Lauri et al., 2014). In addition to its secretory role needed for the development of diverse tissues (for review, see (Stemple, 2005)), the notochord is responsible for neural tube induction. And through the notochord’s secretion of signalling molecules, in particular Sonic hedgehog (Shh), it induces development of the floor plate, situated in the ventral midline of the developing neural tube (Dodd et al., 1998). The notochord and floor plate are intimately linked, and may arise from common progenitors (Gray and Dale, 2010). Both structures secrete Shh which is essential for dorsoventral patterning (Jessell and Dodd, 1990). The Jessell lab was instrumental in furthering our understanding of the role of the notochord, floor plate and resulting Shh in neural development (e.g. (Yamada et al., 1991, Dodd et al., 1998)).

The VZ comprises a population of medial cells along the lumen of the central canal; these cells are in a proliferative progenitor state (Lara-Ramirez et al., 2019). The geometry of this “tube” of progenitor cells is such that the cells are exposed to morphogens, and differentiate based on Cartesian signal gradients (recognising, of course, that this is an oversimplification – see Gouti et al. (2015)). That is, patterning is along the dorsoventral, mediolateral, and rostro-caudal or anterior–posterior (see Lumsden and Krumlauf (1996)) axes (Leung and Shimeld, 2019).

But perhaps the key evolutionary advance for this to occur was Notch signalling (Lara-Ramirez et al., 2019). Notch signalling pathways have several roles in development, including maintaining floor plate progenitors (Latimer and Appel, 2006), and – more significantly for the purpose of this article – in ensuring that the progenitor pool of the VZ is maintained in a proliferative state over developmental time (Appel et al., 2001, Lara-Ramirez et al., 2019). The persistence of progenitor cells leads to a prolonged period of proliferation, and thus an increased number of post-mitotic neurons, and the patterning mechanisms lead to an increase in diversity of neuronal types.

When did this proliferative VZ arise? It is clear that there is a proliferative VZ in the jawed vertebrates (gnathastomes), although there is evidence of earlier precursors. In the lamprey, molecular Notch-regulated mechanisms are similar to those of the jawed vertebrates (Lara-Ramirez et al., 2019). While there are some conserved features from amphioxus, such as Hox-regulated anterior–posterior patterning, there is no VZ in early chordates and dorsoventral patterning does not lead to neuronal diversity with a layered structure of neurons. But it is in the jawed vertebrates that the VZ and thus progenitor cells first appeared and persisted for a period of time, allowing an increased production of post-mitotic neurons. That is, the appearance of the VZ provided a “cohesive progenitor cell pool” that persists through early development and can produce a variety of cell types throughout this period (Leung and Shimeld, 2019).

Thus, two key advances in the early evolution of chordates were the development of the notochord and floor plate that led to neural tube induction and ultimately dorsoventral patterning, followed by the Notch signalling pathway that supported a proliferative VZ. This VZ in turn led to the proliferation of progenitor cells through development. These progenitor cells then provided the means to increase the number of neurons formed, as well as to increase the number of neuronal classes and sub-classes. (Of note, this diversity of neurons generated from dorsoventral patterning formed the basis for a lot of the knowledge that was generated over the decades in the Jessell lab (e.g. Dodd et al., 1998, Ericson et al., 1995, Jessell, 2000)). And it was these numbers and types of neurons that led to the formation of layers of circuits in the spinal cord that ultimately led to an increased repertoire of movement.

## Evolution of the sympathetic nervous system

With this new capacity to generate many neurons, many types of neurons, and layered circuits, came the evolution of the sympathetic nervous system (SNS). Of course, the sensory (dorsal) and motor (ventral) systems of the spinal cord also continued to evolve and develop new microcircuits. But here I will focus on the necessity of the SNS for the evolutionary success of vertebrates.

While the parasympathetic system evolved relatively early, appearing even in invertebrate chordates, the SNS originated later. Since a key role of the SNS is the regulation of cardiac output and blood pressure, it is helpful to first look at cardiovascular evolution.

A continuous endothelial lining of blood vessels (“tubulogenesis”) first appears in vertebrates (in agnathans, ∼510–540 mya, (Shigei et al., 2001)). In contrast, the blood vessels of invertebrates are matrix-lined (Monahan-Earley et al., 2013), although there is evidence of some discontinuous vascular endothelial-like cells in some invertebrates (e.g. octopus and squid (Shigei et al., 2001)). Thus, these invertebrates do not have the capacity to increase their vascular resistance and thus systemic blood pressure. And invertebrate chordates also do not have endothelial tubes (Monahan-Earley et al., 2013). Following the appearance of endothelial lining, vascular smooth muscle appeared, thus providing most vertebrates with 3-layered (intima, media, and adventitia) vasculature (Shigei et al., 2001). These vessels have contractile and resistive properties, and, together with the closed nature of their cardiovascular systems, these animals are able to produce higher systemic blood flow rates and pressures (Monahan-Earley et al., 2013). That is, this structure and resistive properties of the vessels allow energy from blood vessels to add to that of the heart to increase the velocity of blood flow and blood pressure, which depends on blood volume and the interaction of cardiac output and blood vessel resistance.

The evolution of the heart, like that of the spinal cord, relied on the development of antero-posterior signalling and retinoic acid (Simoes-Costa et al., 2005). A precursor of a heart may have appeared in amphioxus – a peristaltic contractile vessel providing low pressure perfusion of the vasculature (Simoes-Costa et al., 2005). Early in vertebrate evolution, the pulmonary circulation (gills) were upstream from the systemic circulation, with the separation of the two evolving in early fishes (Jensen et al., 2013). This was accompanied by increasing septation of the cardiac chambers. Although four-chambered hearts were present in agnathans (Simoes-Costa et al., 2005), full ventricular septation arose first in birds and mammals convergently; these hearts also developed compact muscular ventricular walls that allowed for increases in systemic blood pressures. In addition, the hearts could beat several times faster than earlier vertebrates, which allowed the cardiac output to sustain high systemic metabolic rates (Jensen et al., 2013).

At about the same time that the cardiovascular system was evolving, a CNS system to control the heart and blood vessels was evolving: the SNS (Gaskell, 1916). The SNS is comprised of central, preganglionic neurons and, for the most part, a paravertebral sympathetic chain of ganglia (Kuntz, 1911). There is no evidence of a SNS in amphioxus. In hagfish, although the vessels are endothelial lined (see above), the blood pressure and heart rate are relatively low and the hearts are not innervated. There are no sympathetic chains or segmental sympathetic ganglia in agnathans, where sympathetic-like neurons seem to be distributed along the major veins (e.g. in lamprey and hagfish – these may represent a primitive sympathetic system (Burnstock, 1969)). Interestingly, some transcription factors involved in SNS development in higher vertebrates are present in the lamprey genome, but do not lead to development of sympathetic-like neurons (Haming et al., 2011). In these animals, blood flow in subcutaneous sinuses may be regulated by a subcutaneous plexus (Haming et al., 2011). In elasmobranchs, there are paravertebral ganglia rather than a continuous sympathetic chain, and heart rate is controlled by reflexes from the gill blood vessels (Bagshaw, 1985). In contrast, a sympathetic chain first appears in teleost fish, which can produce higher heart rates (Burnstock, 1969, Bagshaw, 1985, Shigei et al., 2001). In teleosts, the SNS is similar to that in higher vertebrates, with preganglionic and postganglionic fibres associated with a sympathetic chain (Bagshaw, 1985, Nilsson, 2011). That is, a “sophisticated” SNS appeared following the appearance of vascular smooth muscle, and provided neurological control over heart rate and blood pressure.

This meant that instead of relying on a system in which a rising blood pressure leads to a reflex bradycardia, evolution led to a system in which blood pressure could be controlled by the nervous system, and raised or lowered by regulating both heart rate and vascular resistance (Bagshaw, 1985). That is, it seems that the switch in balance from humoral (chromaffin cells) to neural (SNS) regulation led to central control mechanisms of the cardiovascular system (Gaskell, 1916, Shigei et al., 2001).

In summary, the evolution of the VZ led to the development of diverse neuronal types, including those that became the SNS, which developed following the emergence of a layered vascular system. Together, these systems allowed control of cardiac output and blood flow by the CNS. This system was the keystone for the evolution of the combination of power, speed, and endurance in mammals because it led to the emergence of homeothermic endothermy.

## Evolution of endothermy

Homeothermic endothermy was a remarkable evolutionary development and critical to the development of social behaviour. Several ideas about the evolutionary advantages of endothermy have been postulated (see, for e.g., (Grigg et al., 2004)). For example, endothermy allowed animals to occupy thermal niches that were otherwise uninhabitable, thus providing a degree of independence from various environments (e.g. latitudes, altitudes, time of day). Endothermy also led to the young being born at early developmental stages, leading to the opportunities for further maturation in post-natal development under the guidance of a parent. But arguably a principle evolutionary advantage of endothermy was neither thermoregulatory nor parental, but rather to allow the higher rates of aerobic metabolism necessary for increased levels of activity (Bennett and Ruben, 1979).

Homeothermic endothermy arose in birds and mammals, evolving convergently. Interestingly, not all poikilotherms are completely ectothermic – that is, they have regional endothermy (e.g. muscles or eyes or brain) (Block et al., 1993). Some fishes, for example tuna, can maintain their muscles at temperatures higher than the ambient temperature, providing them with the capacity to swim faster and farther (Watanabe et al., 2015). Tuna can swim up to about 70 km/h in bursts lasting 10–20 s (Walters and Fierstine, 1964); that is, they have speed, but do not seem to have endurance (Dickson and Graham, 2004). The fact that regional endothermy arose convergently in teleosts and cartilaginous fish suggests that it provides ecological advantages (Carey et al., 1971), although this is not clear (Dickson and Graham, 2004).

Sustained aerobic metabolism and homeothermic endothermy arose hand-in-hand (Bennett and Ruben, 1979). Aerobic metabolism requires a high rate of oxygen delivery, the rate limiting step in oxygen consumption and aerobic metabolism (Hillman et al., 2013, Hedrick et al., 2015). That is, neither endotherm’s efficient ventilatory systems nor highly-concentrated tissue mitochondria operate at maximum capacity for oxygen uptake or use, respectively. To address the problem of delivering the oxygen to the tissues, a cardiovascular system evolved to efficiently transport blood and oxygen over time and distance, thus allowing increases in both body sizes and metabolic rates (Monahan-Earley et al., 2013). The key requirements were increased blood flow supported by high cardiac output (necessitating high heart rates) and high blood pressure (Hillman and Hedrick, 2015). That is, for endothermy to arise, it was necessary for the cardiovascular system to develop along with the SNS (see above and Fig. 1).

In endotherms, the combination of cardiovascular and SNS development led to the ability to deliver oxygen such that there is a ∼10-fold increase in maximal oxygen consumption compared to ectotherms (Bennett and Ruben, 1979). In other words, endothermy arose on the foundation of cardiovascular and SNS systems, and in parallel with aerobic metabolism, providing the support required for the combination of power, speed, and, in particular, endurance. In contrast, ectothermic animals rely on anaerobic metabolism and can be very fast (consider some reptiles), but this activity occurs in short bursts and is not sustainable.

There is a significant cost to endothermy: in particular, it is energetically expensive and thus requires a significant increase in food intake. In fact, one hypothesis is that endothermy arose due to parental care, which needed increased food intake and metabolism that relied on the function of visceral organs, which are responsible for a high proportion of basal metabolic rate (Koteja, 2000, Farmer, 2000). But the benefit of this high cost was the provision of the ability to sustain high speed locomotor activity, leading to enhanced capacity to defend one’s territory, the ability to increase foraging and hunting, as well as to enhance success in courtship and mating (as is nicely illustrated across Kenya). That is, the evolution of endothermy supported the aerobic metabolism required for a broadly expanded behavioural repertoire that included, in particular, endurance (Bennett and Ruben, 1979, Hedrick and Hillman, 2016).

## A continuous nervous system

Of course, evolution is not so simple as I may have implied above. I have hypothesised that these key steps (Fig. 1) were required to form the platform on which animal behaviour is built. These steps included the evolution of a VZ which resulted in a large number of diverse spinal cord neurons including the SNS. Of course, this system did much more than produce a SNS: rich sensory, motor, and sensorimotor circuits were formed that led to increasingly complex syllables of movement. That is, the cylindrical structure of the spinal cord with its VZ allowed for diffusible molecules during development to set up the cartesian concentration gradients that led to anterior–posterior, dorsoventral, and medio-lateral patterning which resulted in diverse neuronal types. These diverse neuronal types provided the substrate for increasingly complex and differential circuits that led to the expansion and divergence of movement vocabulary. The invertebrate solution to increase movement repertoire (although not the combination of power, speed, and endurance) was to evolve increasingly complex ganglia – a “discontinuous” nervous system. In this final section, I explore whether there are geometrical advantages to a “continuous” spinal cord.

In considering the diversity and number of neurons, it is also interesting to consider the continuity of the grey matter. Although we refer to individual spinal segments based on dorsal and ventral root inputs and outputs, the neurons within the grey matter are distributed beyond segments. Not only do functional units, such as motor pools (but also interneuron pools), spread across multiple segments, but neuronal dendrites themselves spread beyond single segments. Thus, the continuity of functional units across segments allows circuit connections with multiple post-synaptic targets that are not confined to a single segment (cf. invertebrate ganglion). That is, the processing power of a continuous structure of interconnected neurons would be greater than that provided by discrete interconnected ganglia.

Also, the geometry of white matter tracts may be more efficient with a continuous spinal cord: this geometry allows continuous entry and exit of axons, which can travel short or long distances. For example, descending tracts can rapidly transmit signals to the lumbar spinal cord without intervening ganglia. And the same activity can be transmitted to many different segments via axon collaterals resulting in coordination of movement across multiple muscles, joints, and limbs. Thus, spinal cord continuity can enhance the capacity to rapidly effect movements as well as for intralimb and interlimb coordination.

In other words, although this is hard to admit for someone who is electrified by number theory, just as it can be argued that discrete mathematics has its advantages, it can be argued that continuous maths is more efficient at solving problems involving change (consider differential equations). And spinal cord circuits evolved to interact with a continually changing environment, and its continuity may perhaps provide a powerful solution to flexibly solve problems, such as those related to the movements needed for complex social behaviours.

**Fig. 2:**
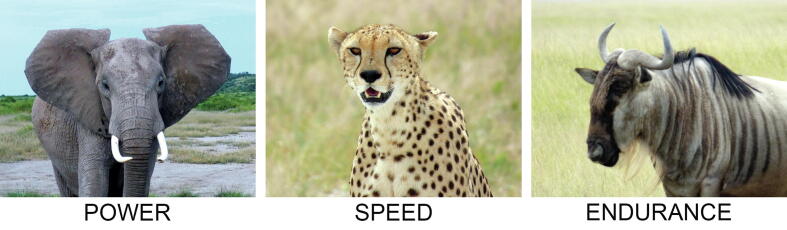
Examples of the success of power, speed, and endurance in Africa.

In summary, I argue here that the evolutionary success of mammals was a direct result of the development of a ventricular zone, sympathetic nervous system, and endothermy. These key steps were the entelechy for a musculoskeletal and nervous system that could support the power, speed, and endurance that are needed to survive and thrive on the African plains (Fig. 2). Movement syllables are programmed by spinal circuits such that descending systems can harness these syllables to provide the languages of movements needed for social and environmental interactions. The increase in neuronal diversity through evolution provided an increase in the syllabic repertoire, which ultimately provided the substrate for the emergence of dexterity.
